# Hand Areas Which Are Commonly Missed during Hand Disinfection by Nursing Students Who Completed a Basic Educational Course in Hand Hygiene

**DOI:** 10.3390/ijerph18052590

**Published:** 2021-03-05

**Authors:** Agnieszka Gniadek, Beata Ogórek-Tęcza, Anna Inglot, Anna Nowacka, Agnieszka Micek

**Affiliations:** 1 Department of Nursing Management and Epidemiological Nursing, Institute of Nursing and Midwifery, Faculty of Health Sciences, Jagiellonian University Medical College, 31-501 Kraków, Poland; agnieszka.gniadek@uj.edu.pl (A.G.); beata.ogorek-tecza@uj.edu.pl (B.O.-T.); anna.nowacka@uj.edu.pl (A.N.); 2 Postgraduate Student at the Faculty of Health Sciences, Jagiellonian University Medical College, 31-501 Kraków, Poland; a.inglot@student.uj.edu.pl

**Keywords:** students nursing, hand hygiene, education

## Abstract

Background: Teaching nursing students how to correctly perform hand hygiene procedures may guarantee a reduction in transmitting pathogens through direct contact and, thus, it may lead to a decrease in the number of hospital infections. The aim of the study, which was conducted in low fidelity simulation conditions, was to assess the performance and the efficiency of a hand-rubbing disinfection technique among nursing students on the last day of their course. Materials and methods: The study was conducted in a group of 190 nursing students studying at the Jagiellonian University and it focused on the performed hand-rubbing disinfection procedure. The accuracy of the task performance was assessed by measuring the percentage of the amount of Fluo-Rub (B. Braun) fluorescent alcohol-based gel remaining on students’ hands after disinfection. The gel was rubbed into particular hand parts including four surfaces (left palm, right palm, left back and right back) divided into thirteen areas (I–XIII) and each surface was examined separately. The results were then dichotomized based on the cut-off point of 10% and two categories: “clean” and “dirty” were established. Additionally, the range of negligence in the disinfection procedure was assessed by counting the total number of the areas classified as “dirty”. The comparison of continuous and categorical variables was conducted by means of Friedman’s and Cochrane’s tests, respectively. Results: It was found out that the palm surfaces that were commonly missed during hand disinfection included the whole thumb (I and VI), the fingertip of the little finger (V) and the midpalm (XIII), whereas in the case of back surfaces (on both right and left hand) the most commonly missed areas were the fingertips and the whole thumb I–VI. Only 30 students (13%) had all 52 areas of both hands completely clean, whereas more than one third—66 students (33%)—failed to disinfect properly more than 10 areas out of all assessed ones on the surfaces of both hands. Conclusions: In the examined group of nursing students, a significant lack of compliance with hand disinfection procedures was observed and it was related mainly to thumbs and back parts of both hands. Therefore, it is essential to conduct systematic training sessions and assessment of hand hygiene procedures for nursing students at the end of every educational stage as it can lead to their developing these skills properly.

## 1. Introduction

Hand hygiene is one of the most important procedures performed in health care institutions by all the staff providing health care to patients as well as by healthy people who visit these facilities in order to receive preventive treatment. Although it is a well-known procedure and precedes every form of medical treatment, it is frequently performed in a careless way, too fast, without the application of proper preparations and also without observing recommended rules (wearing jewellery or clothes which cover wrists). Numerous scientific studies prove that lack of compliance with this simple procedure results in transmitting pathogens to patients and constitute a frequent cause of hospital infections transferred by direct contact [1,2,3,4,5,6,7].

Hand hygiene is a universal term that refers to the application of various methods of decontamination in order to reduce the number of microorganisms that are temporarily present on people’s hands. Hand hygiene procedure in its basic dimension comprises two techniques: hand washing and hand disinfection. The choice of the technique depends on the type of social contact, visible hand soiling, exposure to particular pathogens, possible contact with patients’ mucosa, their discharges, secretions or broken skin [8]. The procedure of hand hygiene that is currently recommended by WHO is called Ayliffe technique and consists of six consecutive steps. In order to effectively stop microorganisms from spreading during a particular sequence of nursing activities, health-care workers are advised to perform hand hygiene procedures following the recommendations defined as “5 Moments for Hand Hygiene” [9,10]. It was proven that after performing the procedure of hand hygiene according to the six steps of hand hygiene, there were still some hand parts that were commonly missed or washed and/or disinfected in an insufficient way. These parts usually include fingertips, thumbs, backs (especially those of index and middle fingers), spaces between fingers and nail areas [11]. Performing hand hygiene according to “5 Moments for Hand Hygiene” is not always implemented while providing health care to patients and the extent to which these rules are observed is estimated at about 40% of actual needs [12,13]. Although the procedure is easy to follow and there are numerous scientific publications recommending it, the extent to which nurses follow these recommendations for hand hygiene procedures still remains below the desired level [3,14,15,16]. The reasons which hinder proper compliance with hand hygiene procedures, and thus, prevent obtaining a high level of proper results include: overburden with care-related duties, limited time to perform this procedure, hand irritation, allergy to chemicals, insufficient supply of disinfectants as well as understaffing and lack of sufficient knowledge, experience and education in this field among medical staff [17,18,19,20].

The review of the previous studies on the methods of evaluation students’ performance of hand hygiene procedures shows that this evaluation is conducted mainly with the application of a diagnostic survey in which the researchers obtained information about students’ knowledge about hand hygiene procedures and their declaration about the skills they possess in this field [21,22,23,24,25,26,27,28,29]. There are also publications that present the findings showing that the ability to perform the hand hygiene procedures in the correct way is verified after educational interventions including theoretical and practical workshops [30] or participation in competitions connected with hand hygiene procedures in which the key element was to prepare efficient educational tools in this area [31]. Additionally, a quantitative and qualitative assessment of the presence of microorganisms on students’ hands was conducted by collecting swabs immediately after the procedure of hand hygiene [32]. A prospective quasiexperiment assessing the influence of the mentor on the indicators of the efficiency of the correct hand disinfection procedure was conducted also among nursing students [33]. The effects of the intervention connected with conventional education within hand hygiene were compared with self-evaluation of students’ own performance based on their observation of fluorescent gel rubbed in their hands [34,35,36]. A systematic review comprising 17 studies on hand hygiene training and educational strategies applied by nurses and nursing students showed that strategies such as reminder sounds, practical simulations, videos, and audiovisual media improved handwashing compliance. These strategies, going beyond commonly practiced educational techniques, e.g., lectures, may be more effective in increasing hand hygiene compliance [37].

Nursing students, while preparing to perform hand hygiene procedure in a proper way, practise it before every practical task in low fidelity simulation conditions within practical undergraduate education. The skill is then developed during further practical tasks in high fidelity simulation conditions and during students’ internship on hospital wards or in other health care facilities. Therefore, before starting providing direct care to patients, they should be able to perform the procedure ideally without any fault so as to take care of patients in a safe way and prevent hospital infections.

The aim of the study was to assess the performance and the efficiency of a hand-rubbing disinfection technique among nursing students in low fidelity simulation conditions on the last day of their course and before direct contact with patients.

## 2. Materials and Methods

It was developed as an observational study conducted in April 2018 in a group of 190 volunteers who were first year nursing students doing their first-cycle course at the Faculty of Health Sciences, Jagiellonian University Medical College. In their eight-semester undergraduate educational cycle, students participate in theoretical and practical classes, including clinical practice. This study was conducted at the end of the second semester of their studies. The time of the study was chosen deliberately as all the students had already acquired theoretical and practical knowledge about hand hygiene and the task that they were supposed to perform in low fidelity simulation conditions was also a part of final credits for their course.

The procedure of hand disinfection both in the theoretical and practical dimension is performed during the first class of the rudiments of nursing course. It belongs to absolutely basic issues connected with the problem of hospital infections (asepsis and antisepsis). This subject does not have an educational effect that would explicitly indicate the necessity to teach students hand disinfection procedures. During further classes of this subject, students focus on the order of the procedure itself (6 steps) and mainly this aspect is emphasized. However, there is no systematic or continuous assessment of its effectiveness.

The inclusion criterium for the study was attending a course in the rudiments of nursing during the first year of the first-cycle nursing studies and a voluntary participation in hand hygiene procedure test. The study was conducted following the recommendation of the Declaration of Helsinki and every student could resign from further participation at any time. The study is a collective report on the results of the test on how students perform hand hygiene procedures which was also a part of final credits for students’ obligatory course within their regular educational cycle. Every student was obliged to perform this task in order to pass the course. The procedure involved the evaluation of the process of education which must be performed by every teacher during the process of students’ education. The description of the findings connected with hand disinfection procedures and obtained during the evaluation of the process of education should be useful for every teacher so as they could improve the efficiency of their didactics, especially during the Covid 19 pandemic. While collecting the data for the manuscript the researchers did not collect any personal data (gender, age, etc.) from students.

Following the publications that estimate that the compliance with hand hygiene recommendations among medical staff ranges between 16% and 81%, 40–50% on average, the required number of participants was calculated. With the expected 55% of students who will perform the procedure in the correct way, the assumption of the two-tailed test, the power of 80% and the level of significance at 0.05, it was calculated that it is enough to examine 188 students in order to reject the hypothesis that the percentage of correctly performed procedures of hand disinfection will reach 65%. The procedure of hand disinfection was supervised by a university teacher—a nurse who was also directly responsible for checking the correctness of performing the procedure.

### 2.1. Study Procedures

**Figure 1:**
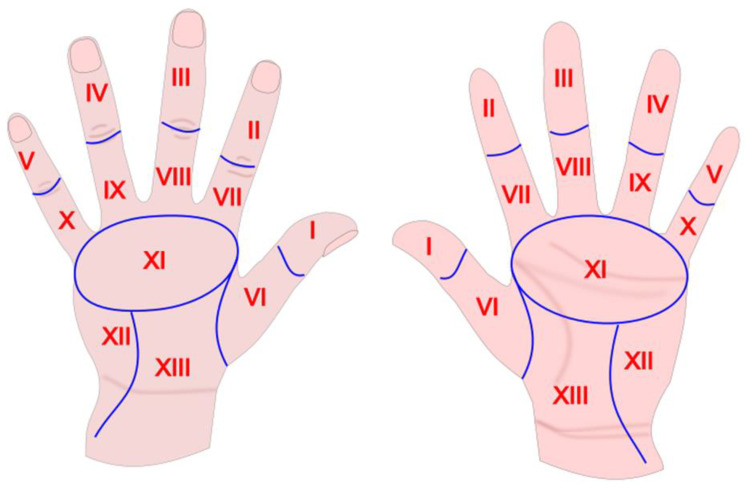
Hand areas.

The participants of the study were asked to perform the procedure of hand disinfection following the required rules as closely as possible according to the European standards accepted in 1997 by the European Committee for Standarization and implemented in Poland in 2002 by the Polish Committee for Standarization (Polski Komitet Normalizacyjny) as PN-EN 1499 and PN-EN 1500 standards. First the students were asked to wash their hands with the application of Ayliffe technique and following PN-EN 1499 standard. Then they went on to the second stage and disinfected their hands by rubbing with the application of Spitaderm disinfectant and following PN-EN 1500 standard. According to the WHO recommendations hand disinfection was performed following six consecutive steps: applying about 3 mL of disinfectant on both hands (in accordance with manufacturer’s instructions), rubbing hands palm to palm, rubbing the back of each hand with the palm of the other one, rubbing the disinfectant in between fingers of both hands, rubbing with interlaced fingers of both hands palm to palm, rubbing both thumbs back and forth alternately left thumb with right palm and right thumb with left palm, rubbing the disinfectant with the fingertips into the palms of both hands and, finally, rubbing the wrists of both hands with back and forth movements. Each step was repeated five times. The standards of the time of washing and hand disinfection procedure were complied with. Afterwards the technique of hand disinfection according to PN-EN 1500 standards (following the same aforementioned procedures) was evaluated by direct observation of Fluo-Rub (B. Braun) fluorescent alcohol-based gel, which was rubbed into particular hand parts [8]. Having completed the disinfection procedure, students put their hands under a portable UV lamp (Black Box) (B. Braun). The device makes it possible to visualize errors in hand hygiene technique owing to a fluorescent dye added to alcohol-based gel, which allows for identification of fluorescent stains on students’ hands (showing which areas were disinfected properly and which were not). As many as 13 areas of both palms and backs of both hands were observed. The supervisor assessed the performance of the task and wrote down the results for every participant of the study assessing the accuracy of the disinfection procedure of both hands (right and left) and both palms and backs. The results were presented as a percentage (from 0 to 100%), where 0 meant a completely clean/disinfected area-Fluo-Rub fluorescent alcohol-based gel was rubbed into the skin and 100 identified totally dirty/not disinfected area–the gel was not rubbed in. The palm and back surfaces of both right and left hand were divided in the same way into 13 areas: fingertips (I–V, where I is a thumb and V is a little finger), lower parts of fingers and the space in between them (VI–X, where VI is a thumb and X is a little finger), space between fingers XI, thenar (XII), midpalm (XIII) (Figure 1).

### 2.2. Statistical Analysis

The assessment of the compliance with hand disinfection procedures was presented by means of continuous variables on the scale from 0 to 100%, where 0 means a thoroughly disinfected area and 100 means a completely dirty area. The percentage of the assessed level of unsuccessful/incorrect hand disinfection was submitted for dichotomy as a result of which two categories: “clean” and “dirty” were established based on the cut-off point of 10% in a basic analysis and to test robustness of the findings on the cut-off point of 30% in a sensitivity analysis. Additionally, the range of negligence during hand disinfection procedure was assessed by summing up the total number of areas which were classified as “dirty”. The total number of the areas missed during hand disinfection procedure (dirty areas) was calculated separately for the back of left and right hand and for the palm of left and right hand (the total ranging from 0 to 13 areas) as well as for the backs of both hands and for the palms of both hands regardless of the side (left or right) (the total ranging from 0 to 26 areas) and finally for both backs and palms of both hands together (the total ranging from 0 to 52 areas). Each obtained sum of “dirty” areas was divided into four categories: 0, 1–2, 3–10 and over 10. The characteristics of all quality variables was presented by means of numbers and percentages. Due to a relatively high number of areas which were classified as “clean” and a significant right-skewed distribution of all continuous variables, they were described as 80, 85 and 95 percentile of distribution and 70, 80 and 90 percentile for backs and palms, respectively. Additionally, for every examined area, the number and percentage of students with completely “clean” and completely “dirty” surface of this area were presented. The significance of differences between the distribution of all continuous and dichotomic variables in all 13 examined areas was checked with the application of nonparametric tests. Bearing in mind that the intensity of hand disinfection measured in 13 different areas of palms and backs of both hands was correlated in the case of a given examined student, the model allowed for the scheme of repetitive patterns. The comparison of continuous and categorical variables was conducted by means of Friedman’s and Cochrane’s tests, respectively. All analyses were conducted with the application of R version 4.0.2 software (Development Core Team, Vienna, Austria) and the values lower that 0.05 were considered to be statistically significant.

## 3. Results

In the case of three examined hand surfaces (palm of the left hand, palm of the right hand, back of the right hand) a statistically significant difference was observed in the distribution of the percentage of incorrect disinfection between thirteen examined areas (I–XIII), whereas the result for the back of the left hand was on the border of statistical significance (*p* = 0.055). It was found out that the surfaces of the palm described as I, V, VI and XIII (the whole thumb area) and the tip of the small finger (V) as well as midpalm (XIII) were the most neglected during disinfection. Additionally, back parts of both right and left hands were the most commonly missed during hand disinfection (Table 1).

**Figure 2:**
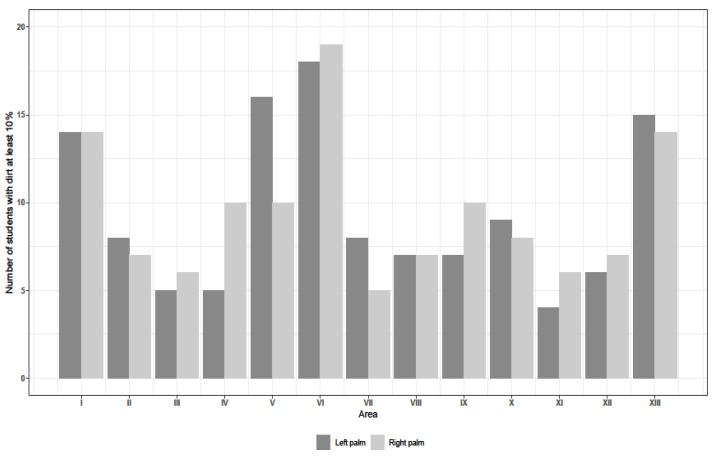
Improperly disinfected palms (dirt ≥ 10%).

**Figure 3:**
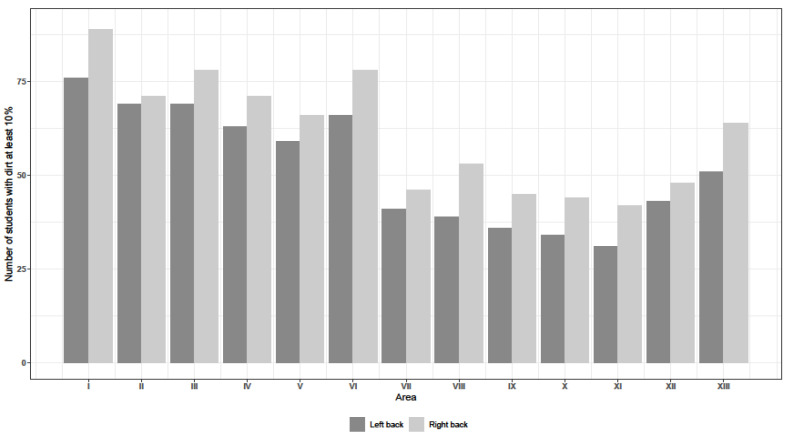
Improperly disinfected backs (dirt ≥ 10%).

A significant difference in the distribution of properly disinfected areas between the thirteen examined ones (the level of dirt <10% vs. ≥10%) was observed on all four examined hand surfaces, which had been disinfected. The comparison of distribution of hand surfaces that were missed during disinfection procedure is presented by Figure 2 and Figure 3 and Table 2. Students tended to disinfect back parts of hands worse than palms and the disinfection of the whole thumb (I and VI) (that is the tip and part I of the thumb and lower part of the thumb–VI) was performed carelessly. Lack of compliance with hand disinfection procedure that resulted in incorrect disinfection on the level of at least 10% was observed most frequently in the following areas: I–thumb fingertips: palms of both hands in the case of 14 students; back of right hand for 89 students and back of left hand for 76 students; VI–lower part of the thumb: palm of the right and left hand for 19 and 18 students, respectively, back surface of the right and left hand for 78 and 66 students, respectively; V–fingertips of a small finger: palm of the right and left hand for 10 and 16 students, respectively, back surface of the right and left hand for 66 and 59 students, respectively as well as XIII–midpalm: palm of the right and left hand for 14 and 15 students, respectively, back surface of the right and left hand for 64 and 51 students, respectively. All in all, students were most likely to miss the areas I, V, VI and XIII. The dirtiest areas on the backs of their hands were I, II, III, IV, V and VI (Table 2).

“Clean”, that is properly disinfected areas (dirt < 10%), were found on students’ palms; that is, in the areas marked as III, XI and XII. Only a small percentage of students failed to disinfect these areas properly and the scale of the reported problems was as follows: five students failed to disinfect III—the tip of the middle finger, four students—XI—space in between fingers, six students—XII—thenar on their left hand, six students—III, six students—XI and seven students—XII on their right hand (Figure 2).

As far as the back hand areas were concerned, the lowest number of students exceeded the dirt level of 10% on their left hands in the areas VII, VIII, IX, X, XI and XII, namely: area VII—41, area VIII—39 students, area IX—36 students, area X—34 students, area XI—31 students and area XII—43 students. In the case of left hand the same trend was observed in the following areas: area VII—46 students, area VIII—53 students, area IX—45 students, area X—44 students, area XI—42 students and area XII—48 students (Figure 3). The sensitivity analysis with the cut-off point of 30% did not change the general conclusions. It showed larger neglects in back parts of the hands compared with palms, and the more frequent omissions of disinfection occurred in the following areas: whole thumb (I and VI), fingertips of a small finger (V), as well as midpalm (XIII).

The most neglected areas on the backs of students’ hands were still I, II, III, IV, V (VI only in the right hand).

Then, it was analyzed in how many areas each student taking part in the study exceeded the 10% level of dirt. The examined ranges included: from 0 to 13 on each of the four hand surfaces separately, from 0 to 26 on each pair of hands (both palms together and both backs together), from 0 to 52 on all four surfaces together (the total of both palms and both backs). Only 30 students (13%) were found to have all 52 areas on both hands completely clean, whereas one third of the students were reported to fail to disinfect properly more than 10 areas out of all examined ones on all four hand surfaces. It is satisfying that as many as 140 students (74%) had properly disinfected palms of both hands and, moreover, 148 students, which means 8 students more than those who properly disinfected palms of both hands in 26 areas, had a thoroughly disinfected one hand and it was equally frequently right and left hand, 148 (78%) vs. 148 (78%). Additionally, 25 students (13%) had only one or two out of 26 areas dirty on palms of right or left hand. A total of five students (3%) had more than 10 dirty areas on palms of both hands. The results for back surfaces were worse as only 34 students (15%) had all 13 areas properly disinfected on both back surfaces (26 clean areas in total) and only in the case of the back of left hand 59 students (31%) disinfected properly all the areas. In the case of right hand the task was successfully completed by only 44 students (23%). Unfortunately, over one third of the respondents—63 students (33%)—failed to disinfect properly over 10 areas on backs of both hands (Table 3). Regarding cut-off point of 30%, the sensitivity analysis showed that 72 students in total (38%) performed disinfection at the level of omission not exceeding 30% in all 52 areas on both hands, whereas 157 students (83%) had clean (<30% of dirt) palms and 76 (40%) had clean backs of both hands (Supplementary Table S2).

## 4. Discussion

The Jagiellonian University is an educational institution that has been incessantly educating nursing students since 1998 within their first-cycle studies and it is also the first university in Poland that started this type of education. Moreover, the course in nursing has gained a prestigious distinction awarded by the Polish Accreditation Committee (Polska Komisja Akredytacyjna), which resulted from the high quality of education on this course. Therefore, the students who graduate from this University with a nursing diploma seem to be adequately prepared for their profession and they should be able to perform procedures correctly, including hand hygiene procedures. First of all, these skills should be properly taught and then developed during practical training in order to reach a satisfying level. These actions should be taken during the first years of medical studies, which is also pointed out by other researchers [38,39,40]. Moreover, the studies show that nursing students have a greater knowledge of hand hygiene procedures than medical students [41,42], which might result from a higher number of didactic hours devoted to teaching these procedures and putting them into practice. Does this higher level of knowledge entail proper practical actions while taking care of patients? This question has been asked by numerous researchers who examined the compliance with hand hygiene procedures among medical staff by assessing both their knowledge and declared behaviours while providing health care services [43,44,45], conducting the observation of these behaviours [46,47,48,49] or assessing indirect indicators of proper hand hygiene procedures including the amount of used hand hygiene supplies [49,50] or, finally, the incidence of hospital infections [4,51].

There are scientific publications that confirm the fact that medical students in Poland still do not master the hand hygiene skills to the extent that would be fully satisfying. One of such studies shows that every fifth student is unable to define even one moment out of “5 Moments for Hand Hygiene” [28]. These results are confirmed by other studies into the knowledge and skills connected with hand hygiene among medical students educated in Poland [25,52,53], as well as in, for example, Slovakia [54] or Germany [38]. This study did not examine, however, students’ declared knowledge and skills connected with hand hygiene, but it tested the actual application of these skills in practice. Taking into account the fact that the study was conducted 7 months after the students started their education in this field and that they had performed the hand hygiene procedure and/or disinfection before every procedure included in their curriculum during these aforementioned months, it was expected that their level of performing the task would be high. Furthermore, the level of students’ awareness of the importance of their compliance with hand hygiene in the context of preventing hospital infections was regularly increased so it might have been expected that the higher level of understanding these rules and procedures should be followed by better and better performing this basic procedure according to the rules. However, this study showed that only 15% of students were able to perform the disinfection procedure in an ideal way (all 52 examined areas on both hands were clean). On the other hand, 74% of students disinfected their palms without any fault. It was also found that the backs of students’ hands were dirty more frequently than their palms, no matter whether it was right or left hand. Both hand backs were disinfected correctly in all the areas by only 18% of students, whereas 74% of students disinfected their palms properly. These results were also confirmed by the study conducted by Scheithauer et al. [38], in which students were also found to disinfect their palms more carefully than their hand backs, just as in the study by Öncü et al. [39], where the assessment of hand disinfection was carried out with the application of the same methods as in this study. What may be the matter of great concern is the fact that 33% of students, that is every third student completing their basic education in hand hygiene, did not perform correct disinfection in at least 10 areas out of all the examined ones (52). This observation is not an estimable one taking into account the assessment of the effects of education provided to students within the whole two-semester cycle. The question arises why the students who know the hand hygiene rules and perform the procedure regularly during all their classes, still do it in an unsatisfactory way. In our opinion, these results should be interpreted not as a failure within educational skills but as information as to which elements of education should be corrected and developed in the further cycles. As it is observed by various researchers [38,42] the control over the correctness of performing hand cleaning or disinfection is not always maintained and hand hygiene as a procedure is not always reinforced as a desired situation during the whole training for nursing profession [55]. Perhaps some other methods of education or verification of the effects of education should be found, a good example of which might be the application of stimulation methods involving so called standardized patient [56]. It seems that introducing control standards for performing hand hygiene procedures and having them assessed regularly, for example, every month, might bring about a change and raise the understanding of the importance of the procedure in question.

In scientific publications dealing with this topic, the information may be found that the areas that are frequently missed while washing or disinfecting hands are fingertips, hand backs (especially index and middle fingers), the space in between fingers and the area around nails [8,11,57]. This study also showed that students additionally failed to disinfect properly thumbs of both hands, fingertips, especially on finger I on both surfaces and fingers I–V on their backs. These results coincide with the results obtained in the studies conducted by Scheithauer et al. [38] Öncü et al. [39] and Szilágyi et al. [58]. The thumbs are not properly disinfected or most frequently missed during the whole disinfection procedure performed by students, who additionally perform the procedure carelessly especially on the back of this finger. Comparable findings were obtained in Turkish studies in which also the dirtiest areas were spaces between fingers and fingertips (35). In the context of obtained results it seems worthwhile to pay special attention to the procedure of rubbing the thumb while teaching hand hygiene skills because any negligence on the basic level and not paying enough attention to this step while applying disinfectant may implicate further failures to comply with these procedures during further education or professional work. What is more, proper thumb disinfection is extremely important as it is the finger that plays a crucial role in numerous medical procedures along with the fifth finger (which also tended to be insufficiently disinfected).

This study, allowing for exceeding the 10% limit of “dirt” in maximum 2 out of 52 disinfected areas of 4 hand surfaces, showed that 80 students (42%) performed disinfection in the correct way. Such a result is placed lower than the initial expectations that 65% of students should perform the disinfection procedures correctly. Similarly, Ceyalan et al. [35] claimed that regardless of nursing students’ positive perception of hand hygiene procedures and their good routines, their hand hygiene compliance is still low. According to numerous scientific studies hand hygiene (and especially its correct performance) has an influence on an increase in the number of incidents in which infectious factors are transmitted, and first and foremost, on the number of hospital infections recorded while providing health care services to patients [4,43,46,49,51,59]. However, there is no standard method which would assess the adherence of the procedure to WHO recommendations [8]. Therefore, in order to limit transmitting infections it is vital to continuously monitor hand hygiene on every stage: while training students for their job, during their postgraduate or specialist education or while performing professional tasks and providing health care to patients. Medical staff must perceive hand hygiene, and especially disinfection, as an essential element of controlling Healthcare Associated Infections (HAI), which pose one of the most important challenges for healthcare systems all over the world. The problem of HAI is complex and multicausal but proper hand hygiene among medical staff is a chance for its successful prevention [60]. Numerous studies show a direct connection between the HAI level and the level of performing proper hand hygiene among medical staff [61]. Professional education of nursing students is aimed at learning and acquiring competences that are indispensable in their future job, and, therefore, they should be given enough time for learning and developing proper attitudes and routines [62,63]. It may be difficult, however, because in the case of the rudiments of nursing, the course syllabus does not include in the effects of acquiring particular skills a specific effect referring directly to performing the hand hygiene procedure. Therefore, it can lead to the impression that this resulting activity is not perceived as particularly important in the process of students’ education. This conclusion is confirmed by the study conducted in Slovakia, which also showed that an analysis of the content of educational programs for nurses reveal significant deficits in the quality and the amount of information connected with hand hygiene [54].

Moreover, nursing education should increase students’ independence, autonomy, motivation, responsibility for patients and awareness of the targets and dangers related to undertaking particular actions connected with patients which are based on acquiring and implementing theoretical and practical rules of hand hygiene.

To sum up, a strong point of our study was obtaining a scientific/didactic proof confirming that it is necessary to introduce some changes in the syllabus of the rudiments of nursing course. These changes should refer to continuous control over this skill and, even more importantly, to raising students’ awareness and motivation to comply with the procedures and their self-evaluation of correct performance of hand disinfection which can be assessed by means of fluorescent gel rubbed in hands. A weak point of the study is its single character and lack of possibility to compare verified skills between, e.g., the first and the second semester of education. It indicates the necessity to monitor students’ progress in hand hygiene procedures not only at the end of the course but continuously, e.g., once a month.

## 5. Limitations of the Study

Let us move on to the limitations of this study. There was no second examination of the compliance with the disinfection procedures, for example, after the third year of studies just before students’ graduation or after completing their internship, which would make it possible to verify the quality of performing these procedures by students just before their starting work in medical facilities. The studies should also be conducted at other universities that run nursing courses so as to observe if students at other universities make the same mistakes as those at the university in Krakow, which would help to answer the question whether the classes which should develop these skills are run efficiently everywhere for all nursing students taking into account the fact that higher education in Poland is based on common educational standards announced by the Minister of Science and Higher Education. Nevertheless, the findings of the study may contribute to further research in this field and, first and foremost, may be the basis for showing the advantages of the application of such a method of monitoring students’ progress in their process of education.

## 6. Conclusions

1. The method of assessing the quality of disinfection applied in this study makes it possible to assess the correct performance of this procedure among nursing students.
2. In the examined group of students significant problems in performing the procedure of hand disinfection were observed and they were connected mostly with thumbs of both hands and backs of both hands.
3. It seems legitimate that regular training and assessment of the compliance with hand hygiene procedures should take place after completing each cycle of nursing education and special attention should be paid to controlling if the person responsible for training and assessing students also performs hand hygiene procedures in the correct way.

## Figures and Tables

**Table 1:** Descriptive statistics of the frequency with which the examined thirteen areas of the four hand surfaces are missed by students.

Statistics	Area of the Hand													
	I *n* = 190	II *n* = 190	III *n* = 190	IV *n* = 190	V *n* = 190	VI *n* = 190	VII *n* = 190	VIII *n* = 190	IX *n* = 190	X *n* = 190	XI *n* = 190	XII *n* = 190	XIII *n* = 190	*p* *
Palm of the left hand														
completely clean, *n* (%)	171 (90)	179 (94)	182 (96)	182 (96)	169 (89)	168 (88)	179 (94)	180 (95)	179 (94)	177 (93)	186 (98)	184 (97)	175 (92)	
completely dirty, *n* (%)	6 (3)	5 (3)	4 (2)	4 (2)	9 (5)	6 (3)	4 (2)	4 (2)	4 (2)	8 (4)	4 (2)	4 (2)	5 (3)	
percentiles 90 (85–95)	0 (0–42)	0 (0–5)	0 (0–0)	0 (0–0)	5 (0–72)	5 (0–40)	0 (0–5)	0 (0–3)	0 (0–5)	0 (0–5)	0 (0–0)	0 (0–0)	0 (0–28)	0.0201
Palm of the right hand														
completely clean, *n* (%)	173 (91)	181 (95)	181 (95)	179 (94)	178 (94)	169 (89)	183 (96)	181 (95)	179 (94)	180 (95)	184 (97)	183 (96)	174 (92)	
completely dirty, *n* (%)	8 (4)	6 (3)	5 (3)	7 (4)	7 (4)	7 (4)	4 (2)	4 (2)	5 (3)	5 (3)	4 (2)	3 (2)	5 (3)	
percentiles 90 (85–95)	0 (0–78)	0 (0–0)	0 (0–0)	0 (0–8)	0 (0–8)	6 (0–58)	0 (0–0)	0 (0–0)	0 (0–8)	0 (0–3)	0 (0–0)	0 (0–0)	0 (0–21)	0.0455
Back of the left hand														
completely clean, *n* (%)	106 (56)	112 (59)	113 (59)	119 (63)	122 (64)	117 (62)	143 (75)	145 (76)	147 (77)	149 (78)	154 (81)	146 (77)	134 (71)	
completely dirty, *n* (%)	29 (15)	26 (14)	30 (16)	29 (15)	30 (16)	4 (2)	24 (13)	24 (13)	19 (10)	22 (12)	20 (11)	16 (8)	18 (9)	
percentiles 80 (70–90)	52 (20–100)	32 (16–100)	35 (17–100)	36 (15–100)	40 (11–100)	20 (15–36)	10 (0–100)	10 (0–100)	5 (0–82)	5 (0–100)	0 (0–100)	10 (0–66)	20 (0–81)	0.0548
Back of the right hand														
completely clean, *n* (%)	89 (47)	112 (59)	106 (56)	115 (61)	119 (63)	108 (57)	136 (72)	130 (68)	136 (72)	139 (73)	147 (77)	141 (74)	123 (65)	
completely dirty, *n* (%)	37 (19)	34 (18)	33 (17)	32 (17)	32 (17)	16 (8)	33 (17)	33 (17)	25 (13)	30 (16)	26 (14)	25 (13)	32 (17)	
percentiles 80 (70–90)	91 (37–100)	65 (20–100)	72 (30–100)	50 (20–100)	50 (15–100)	30 (15–90)	16 (0–100)	51 (5–100)	11 (0–100)	10 (0–100)	16 (0–100)	20 (0–100)	36 (15–100)	0.0001

* from Friedman test.

**Table 2:** Distribution of students with dirt at level at least 10%.

	Palm of the Left Hand (*n* = 190)	Palm of the Right Hand (*n* = 190)	Back of the Left Hand (*n* = 190)	Back of the Right Hand (*n* = 190)
Area	Dirt ≥ 10%	Dirt ≥ 10%	Dirt ≥ 10%	Dirt ≥ 10%
I	14 (7.37)	14 (7.37)	76 (40)	89 (46.84)
II	8 (4.21)	7 (3.68)	69 (36.32)	71 (37.37)
III	5 (2.63)	6 (3.16)	69 (36.32)	78 (41.05)
IV	5 (2.63)	10 (5.26)	63 (33.16)	71 (37.37)
V	16 (8.42)	10 (5.26)	59 (31.05)	66 (34.74)
VI	18 (9.47)	19 (10)	66 (34.74)	78 (41.05)
VII	8 (4.21)	5 (2.63)	41 (21.58)	46 (24.21)
VIII	7 (3.68)	7 (3.68)	39 (20.53)	53 (27.89)
IX	7 (3.68)	10 (5.26)	36 (18.95)	45 (23.68)
X	9 (4.74)	8 (4.21)	34 (17.89)	44 (23.16)
XI	4 (2.11)	6 (3.16)	31 (16.32)	42 (22.11)
XII	6 (3.16)	7 (3.68)	43 (22.63)	48 (25.26)
XIII	15 (7.89)	14 (7.37)	51 (26.84)	64 (33.68)
χ^2^	55.7	40.6	141.6	136.6
*p* **	<0.001	<0.001	<0.001	<0.001

Results are expressed as *n* (%); ** *p*-values refer to Cochrane test.

**Table 3:** The number of areas which were not disinfected properly by students.

Total Number of Areas with Dirt ≥ 10%	0	1–2	3–10	>10
Palm of left hand, areas I-XIII	148 (78)	32 (17)	6 (3)	4 (2)
Palm of right hand, areas I-XIII	148 (78)	30 (16)	9 (5)	3 (2)
Back of left hand, areas I-XIII	59 (31)	48 (25)	63 (33)	20 (11)
Back of right hand, areas I-XIII	44 (23)	54 (28)	64 (34)	28 (15)
Both palms of hands, areas 2x (I-XIII)	140 (74)	25 (13)	20 (11)	5 (3)
Both backs of hands, areas 2x (I-XIII)	34 (18)	50 (26)	43 (23)	63 (33)
Both palms, backs of hands, areas 4x (I-XIII)	30 (16)	50 (26)	47 (25)	63 (33)

Results are expressed as *n* (%); 0—number of clean areas, 1–2, (one or two areas with dirt), 3–10 (from 3 to 10 areas with dirt), >10, more than 10 areas with dirt.

## Supplementary Materials

The following are available online at https://www.mdpi.com/1660-4601/18/5/2590/s1, Table S1: Distribution of students with dirt at level at least 30%, Table S2: The number of areas which were not disinfected properly by students.
