# Supplementary material for: Hand Areas Which Are Commonly Missed during Hand Disinfection by Nursing Students Who Completed a Basic Educational Course in Hand Hygiene
Source: Int J Environ Res Public Health. 2021 Mar 5;18(5):2590. doi: 10.3390/ijerph18052590 (PMC7967523; doi:10.3390/ijerph18052590)
Supplement: Supplementary file 1 [file ijerph-18-02590-s001.pdf]

Supplementary Table S1. Distribution of students with dirt at level at least 30%

Area	Palm of the left hand (N = 190) dirt ≥ 30%	Palm of the right hand (N = 190) dirt ≥ 30%	Back of the left hand (N = 190) dirt ≥ 30%	Back of the right hand (N = 190) dirt ≥ 30%
**I**	11 (5.79)	12 (6.32)	55 (28.95)	65 (34.21)
**II**	7 (3.68)	6 (3.16)	45 (23.68)	55 (28.95)
**III**	4 (2.11)	5 (2.63)	49 (25.79)	58 (30.53)
**IV**	4 (2.11)	8 (4.21)	46 (24.21)	54 (28.42)
**V**	13 (6.84)	7 (3.68)	47 (24.74)	46 (24.21)
**VI**	13 (6.84)	13 (6.84)	27 (14.21)	45 (23.68)
**VII**	6 (3.16)	4 (2.11)	32 (16.84)	37 (19.47)
**VIII**	5 (2.63)	5 (2.63)	32 (16.84)	44 (23.16)
**IX**	4 (2.11)	6 (3.16)	29 (15.26)	33 (17.37)
**X**	8 (4.21)	6 (3.16)	31 (16.32)	37 (19.47)
**XI**	4 (2.11)	6 (3.16)	27 (14.21)	36 (18.95)
**XII**	5 (2.63)	5 (2.63)	30 (15.79)	34 (17.89)
**XIII**	10 (5.26)	7 (3.68)	30 (15.79)	45 (23.68)
χ^2	46.1	28.9	77.3	85.1
p^{**}	<0.001	0.004	<0.001	<0.001

Results are expressed as n (%); **p-values refer to Cochrane test.

Supplementary Table S2. The number of areas which were not disinfected properly by students

Total number of areas with dirt ≥ 30%	0	1-2	3-10	>10
**Palm of left hand, areas I-XIII**	161 (85)	22 (12)	3 (2)	4 (2)
**Palm of right hand, areas I-XIII**	163 (86)	19 (10)	5 (3)	3 (2)
**Back of left hand, areas I-XIII**	92 (48)	38 (20)	44 (23)	16 (8)
**Back of right hand, areas obszary I-XIII**	89 (47)	36 (19)	40 (21)	25 (13)
**Both palms of hands, areas 2x (I-XIII)**	157 (83)	16 (8)	13 (7)	4 (2)
**Both backs of hands, areas 2x (I-XIII)**	76 (40)	32 (17)	41 (22)	41 (22)
**Both palms, backs of hands, areas 4x (I-XIII)**	72 (38)	31 (16)	45 (24)	42 (22)

Results are expressed as n (%)
